# Community-Based Qualitative  Study to Identify Challenges in Implementing Health Promoting Schools Framework in Government Schools of Khordha District, Odisha, India

**DOI:** 10.7759/cureus.41872

**Published:** 2023-07-14

**Authors:** Aravinda Chinnadurai, Arvind K Singh, Binod K Patro, Binod K Behera

**Affiliations:** 1 Department of Community and Family Medicine, All India Institute of Medical Sciences, Bhubaneswar, Bhuabneswar, IND; 2 Department of Community Medicine, Government Medical College, Ariyalur, IND; 3 Department of Community and Family Medicine, All India Institute of Medical Sciences, Bhubaneswar, Bhubaneswar, IND

**Keywords:** health policy, who, adolescents, school health, health promotion

## Abstract

Background: The WHO asserts every school should be a Health Promoting School (HPS) and laid a framework. The WHO is constantly working on expanding the number of schools modelled on it. The status of the level of health promotion needs to be explored to understand the local issues to identify key priority areas for policy making towards positive health. The study aims to explore the challenges in implementing the HPS framework in Government-run schools in Odisha.

Method: A qualitative study was conducted with grounded theory using an inductive approach. In-depth interview was done among nine teachers from six government schools in the Khordha district who were selected through purposive sampling. A semi-structured open-ended interview was conducted using an interview guide among school science teachers and principals (n=9) regarding the challenges in implementing the HPS framework. Codes were generated from the transcript using the inductive approach from the WHO-HPS framework. Thematic analysis by Braun and Clarke model was using Quirkos software.

Results: Five themes with 12 codes were identified with the transcript. The respondents had little knowledge about the WHO-HPS framework. Their perception of health promotion was restricted to organizing health camps for school children. The themes were School health policy (inconsistent teachers recruitment policy, partial implementation of tobacco-free schools, outdated science syllabus), Coordination with the local community, Healthy school needs (lack of interactive and repetitive health-related training for school teachers, first aid box in schools and lack of supplementation of micronutrients like WIFS), Sanitation (inadequate funding for maintenance of sanitation) and an emerging issue of increased use of social media were reported in our findings.

Conclusion: Our findings suggest that implementation of health-promoting schools requires imparting the skills to teachers by orientation and expanding the existing health services, backed with adequate funding and a firm policy commitment at the state level.

## Introduction

Schools create a unique hope to improve children's education and health status. The WHO defines a Health Promoting School (HPS) as "a school that is constantly strengthening its capacity as a healthy setting for living, learning and working." ​WHO defined six key characteristics or “pillars” of HPS: healthy school policies, healthy physical school environments, healthy school social environments, health skills and education, links with parents and the school community, and access to school health services [1]. The HPS framework encompasses the broad spectrum of health promotion that includes health policy, rendering health services, strengthening community participation, and creating supportive environments [2].

A health-promoting school enables pupils to have good social and emotional health and improved academic learning. Schools with an established health policy, defined curriculum, maintenance of sanitation and environmental hygiene, enabler of physical activity, psychosocial safety, cooperative learning, group cohesion, behavioural and emotional engagement, and better cooperation from the local community are associated with a positive climate ​[3-6].

In India, the state governments cater to the community's health and education needs. However, the Union government frames guidelines and policies and provides partial funds for the education and health of the states. In India, there is no uniform policy regarding health promotion in schools. School education in the Indian system follows a mixed model where public and private schools function simultaneously. The proportion of students studying in private schools gradually rose to 50% in 2021 [7]. Only recently, under the Ayushman Bharat program, the Government of India published operational guidelines based on inputs from WHO in 2018 to promote the health and well-being of school children. The Odisha state government currently conducts a school health program (RBSK), a centrally sponsored program to reduce childhood morbidity [8].

With the world's largest youth population, India requires an inspiring demographic dividend that needs to have a lasting impact on children. The status of the health promotion level needs to be studied to understand preexisting, neglected, and emerging issues. The qualitative assessment of health promotion status is vital to identify key priority areas ​[9-10]. 

Hence the present study was carried out to explore the challenges in implementing the health-promoting school's framework in the context of the HPS framework of the WHO in the Khordha district of Odisha, India.

This study aims to explore qualitative data unique and specific to the government-run schools in the eastern Indian state of Odisha. It included evaluating the perception of schools' health promotion by educators, availability of funding for maintenance of sanitation, examination of the curriculum, emerging issues, issues with the organogram of the school education department, implementation of legislation, and issues related to the provision of emergency medical services.

Research questions for the current study are: (1) What practical issues did they face in implementing health promotion? (2) How the local community interacts with the school administration? (3) How do they maintain sanitation and the factors involved in it? (4) How does the school handle health and emergencies? (5) What are the emerging issues that need attention in the future?

## Materials and methods

Methodology 

A qualitative study using grounded theory was done from Jan 2021 to April 2021 to explore perceived challenges by school teachers in implementing the HPS framework. The study was done in government-run high schools of field practising areas of the community and family medicine department of All India Institute of Medical Sciences, Bhubaneswar. The areas include the rural areas of Mendhsal block and Tangi and the urban areas of Nayapalli in Bhubaneswar. The study participants were the school administration and teachers at selected government schools in the Khordha district. Permission was obtained from the Institute Ethics Committee at All India Institute of Medical Sciences, Bhubaneswar (AIIMS/PG/Thesis/2019/57) and District Education Department authorities (Memo no: 11381, Dated 11.12.2020) for the study. Consent was obtained from the participating schools after providing the participant information sheet.

The schools were initially assessed quantitatively based on a checklist prepared separately (not a part of this study). The time was used to get a formal introduction and acted as an icebreaker to build rapport between the interviewer and the participants. Then purposive sampling was done to select the schools for qualitative study. The school principals who could afford the time and experience and were perceived as knowledgeable by the interviewer were approached to interview for the qualitative inquiry. The study was partially financially supported by the Indian Council of Medical Research (ICMR). 

The first author, a male working as a junior resident, conducted the interview, pursuing post-graduation in MD Community Medicine. The interviewer worked for four years as a medical officer working closely with the school health department in different districts. The interviewer's past engagement was in a different district, and the interviewer received training for qualitative research before the study. Before the commencement of the study, an online introduction section was organized on Google Meet to brief the stakeholders regarding the aim and purpose of the study. The participants were informed about the academic credentials of the first author in the session.

The interview guide was prepared from WHO guidelines for the HPS framework, and local context was added through informal discussions with district-level school administrators [9]. The interview guide was pilot tested by conducting a semi-structured interview with two teachers who were not part of the study participants. The interviewer guide is attached as appendices in Table 1.

**Table 1:** Interview guide used for the study after pilot testing

Interview Questions	Prompts
The challenges you feel about providing/facilitating health services like immunisation and IFA tablets.	Are there any adverse effects among children? How is the information provided by the health worker helpful?
Challenges faced that are exclusive to Girls only/boys only/Co-ed schools.	Any specific behaviour among boys or girls within their cohort?
Challenges faced by school teachers in making decisions faced during emergency cases.	Have you ever faced any emergency? How you handled it during that time?
Support by GoO in getting physical infrastructure.	How would you rate the standards of the schools? What are your expectations in terms of physical infrastructure?
Difficulty in managing defaulters.	How do you tackle the adolescent aspect behaviours of the children?
Mobile using behaviour among school students.	Are students using mobiles? Are students using social media on mobiles?
Tobacco and liquor-related behaviours	Are students aware of the harmful effects of tobacco and alcohol? Are they using it?
Cooperation of parents	How involved are parents in the day-to-day academics and health of their children?
Utility of training received respective to health.	How would you rate the training given to you? How relevant are they for school health needs? Do you have any suggestions for improvement in the delivery of training?
Availability of funds for health-related activities	Do you have any funds for spending on health-related activities? If insufficient, how much extra would you need?

The newer areas of interest arising during the interview were subsequently added. Semi-structured interviews with selected school teachers and principals collected the qualitative data. The interview was conducted face-to-face inside the school campus. There was no drop out from the study.

The data collection was done until we reached saturation, where no new information or themes evolved. When used in the broader context, saturation refers to the point in data collection when no additional issues or insights are identified, and data begin to repeat so that further data collection is redundant, signifying that an adequate sample size is reached [11]. We employed theoretical saturation during sampling and inductive thematic saturation during analysis to check for the adequacy of the sample size [12]. 

The HPS framework includes the school curriculum teaching health topics (usually taught in the science syllabus), and the health and wellness ambassadors were usually science teachers in the schools. Hence school principals and science teachers were interviewed individually as participants in their respective schools with the interview guide. The purpose of the qualitative assessment was explained in detail to the participants. The interview was conducted in English, and the interview duration ranged from 25 to 40 minutes. The participants were informed that the data collected would be anonymized and kept confidential, and the identity of the participants would not be shared with the higher authorities. Data was collected from nine teachers from six schools. There are no dropouts in our study. To ensure that the district education authorities were not involved in selecting the schools for the interview and that the school list was prepared by the first author from the list of schools provided by the district authorities. 

The transcripts were noted down in handwritten notes in Microsoft OneNote. After completing the interview, the notes were shown to the participants to confirm the data. Later the data were copied into an MS Word document.

The authors read the transcript multiple times using an inductive approach to identify recurring themes and similar patterns. It was coded independently by the first author and the second author and then imported into the Quirkos 2.4.1 software (https://www.quirkos.com/index.html). From the word cloud generated and the multiple readings recurring words and themes were identified and noted. Since recurring themes arose, it was decided to do a thematic analysis of the qualitative content. Selected quotations were identified and inserted in a code. The data were then coded, and thematic analysis was done. Coreq checklist was used and summarised in Table 3 in the Appendix [13].

## Results

**Figure 1:**
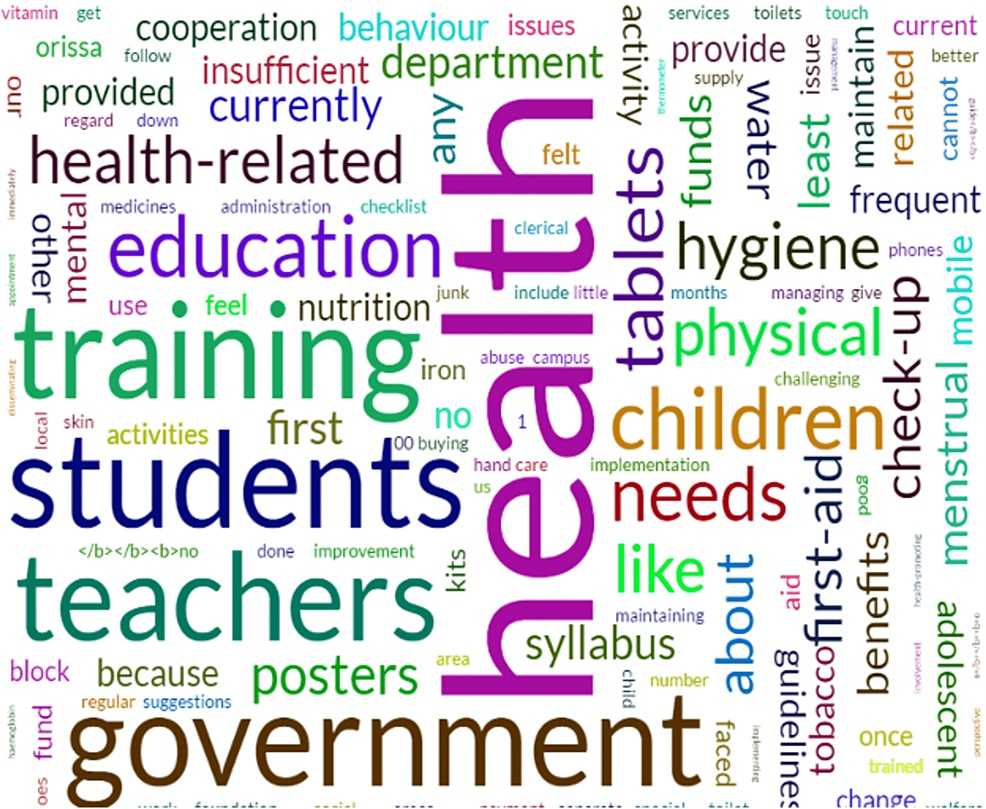
Word cloud generated from the transcript using the Quikos software

A total of nine participants were included in the in-depth interview. The interview was conducted in six schools. The school principals had a minimum experience of 15 years; four were male out of six, while all three science teachers were female, with a minimum experience of two years. Since the concept of health promotion is unfamiliar to them and the school principals and teachers comparing homogenous categories, saturation was reached within nine interviews held separately with the participants in 30 to 40 minutes per participant. So the saturation of data was reached with the limited sample. Five themes and 12 codes were generated, as shown in Table 3. A word cloud was generated from the Quikos software, as shown in Figure 1.

**Table 2:** Thematic framework of issues in health promoting schools

Sl.no	Codes	Themes
1	Block Grant Schools	1. Government of Odisha policies
2	School Syllabus	
3	Anti-punishment Policy	
4	Tobacco-free Schools	
5	Cooperation of Parents	2. Cooperation of the community
6	Cooperation of Local body members	
7	Sanitation	3. School Environment
8	Physical Infrastructure	
9	School Health Financing	4. Healthy School Needs
10	Health Services Availability	
11	Health-related training	
12	Mobile and Social Media Usage	5. Emerging issues

Perceptions of health promotion in schools 

All the participants acknowledged the importance of promoting health in schools. However, their idea of health promotion is mainly restricted to organizing health camps. The participants perceived that the Government of Odisha gives very little importance to health promotion. The respondents did not have much idea about the World Health Organization Health Promoting Schools framework.

Theme 1: Government of Odisha policies 

Code 1: Block Grant Schools 

The participants said that the Government of Odisha policies should be proactive in facilitating school health promotion. The schools aided by the Government, known as "Block grant schools/Govt aided schools" were observed to lack all aspects, specifically the environmental component. The school teachers in these schools were not paid at par with other teachers. The school children do not receive specific welfare schemes like free books, uniforms, and midday meals. 

"The school is a block grant school. It is not at all taken care of by the Government of Odisha. The school does not even have the facility for toilets for boys and girls, drinking water, and uniforms for school children." {Principal School 1, Male, age 51}.

"The students studying in the block grant school are not getting the benefits that an average child will get in schools run by funded by the education department. For example, the school children do not receive uniforms, cycles, books, mid-day meals, and other benefits they will get in other schools." {Principal School 1, Male, age 51}.

*Code 2: School Syllabus* 

The science teachers were interviewed for this purpose. They believed the syllabus was boring and theoretical and had little practical value. Another criticism they levelled at the syllabus, it is too bulky and outdated.

"The syllabus covering health-related subjects is theoretical and has little practical value". {Science teacher School 2, female, age 40} 

*Code 3: Anti-Punishment Policies* 

The Government has banned any forms of punishment for school students. Though all the selected schools seemed to be committed to implementing the anti-punishment policy, they found it very challenging to maintain discipline inside the school campus. 

"Ban for giving punishments to students is particularly challenging to maintain. It is also an obstacle for maintenance of discipline and to regulating the school students." {Principal School 4, female, age 55}.

Code 4: Tobacco-Free Schools 

The respondents reported that implementing tobacco-free guidelines requires cooperation from local bodies, especially the Local body members. Teachers can only monitor school students during school hours and prevent them from consuming tobacco/liquor during school hours only. 

"To effectively implement tobacco-free school guidelines as per the national guidelines, we need open cooperation and active involvement of local body members. Unfortunately, however, this happens very rarely." {Principal School 6, Male, age 53}.

"At best, we can control the usage of tobacco and liquor-related products inside the school campus during their school timings only." {Principal School 2, female, age 50}.

Theme 2: Cooperation of parents and local body leaders 

*Code 5: Cooperation of Parents* 

Parents of the children admitted to these schools were illiterates, daily labourers below the poverty line. They were least bothered about the improvement of their children. At times, they even ignore the serious health issues of their children. They do not play any meaningful role in initiating health-related activities, but some participate in the events.

"Parents of the children in our school are least bothered about their children once they send them to school. This behaviour has to change, and they should have some sense of responsibility for their children." {Principal School 3, Male, age 48}.

Code 6: Cooperation of Local Body Members 

This perception largely varied from school to school. Schools rated them as very cooperative - ignorant - troublesome depending upon the local body members. The cooperation depends upon the cordial relationship between the individual relationship between the headmaster and local body members.

"Sarpanch cooperation needs to ensure removing tobacco or alcohol shops situated nearby. In my capacity, I am not allowing the school students outside it during school time, which prevents students from accessing those shops." {Principal School 6, Male, age 53}.

Theme 3: School environment and sanitation 

*Code 7, 8: Sanitation and Physical Environment* 

Maintaining sanitation and hygiene is particularly challenging in government schools, as reported by the respondents. Many of them cited a lack of specific allocation of financial resources. In block grant schools, the situation is very worst. Even basic amenities like toilets and hand washing facilities were not available.

"There is an insufficient number of urinals in the toilet." {Principal School 3, female, age 48}.

"Specific grants need to be provided to maintain cleanliness in toilets, and infrastructure needs to be provided for dining facilities." {Principal School 5, Male, age 57}.

"Pure drinking water is an issue." {Principal School 6, Male, age 53}.

"Each student bringing water bottles and tiffin boxes would help maintain better personal hygiene and the environment." {Principal School 6, Male, age 53}.

Theme 4: Healthy schools needs 

Code 9: Health Services Availability 

The schools do not have any established protocol for first aid, and the first aid boxes of many schools were not in place and sometimes contained expired medicines. 

"In addition, there should be a separate allocation of funds for first-aid kits, or the Department of school education should provide first-aid kits for individual schools." {Science teacher School 3, female, age 31}.

"Along with iron tablets, calcium and vitamin tablets should also be given since most students are underweight." {Science teacher School 2, female, age 40}.

*Code 10: School Health Financing* 

There are no separate decentralized financial provisions at the school level. There is a provision of ₹75000 per annum for schools given to the School Management and Development Committee. However, this seems inadequate, mainly for High and secondary schools. 

"School maintenance fund currently for Rs.50,000 is inadequate. It should be at least 1.5 lakhs per annum." {Principal School 5, Male, age 57}.

"The government also needs to give us sufficient funds to spend on basic needs such as maintaining sanitation, repairing water supply works, buying medicines for first-aid, maintaining playgrounds, and buying sports equipment." {Science teacher School 2, female, age 40}.

*Code 11: Health Training* 

On inquiry, it was learned that the teachers received many health training from the state health department. The training types include first-aid training, mental health training, "Khushi" training, life skill education training, eye check-up training, Children With Special Needs training, adolescent health training, AIDS awareness training, and COVID-19-related training for the pandemic. The Department of School Education conducts most of the training. The teachers who received training desired follow-up training and preferred offline over online. 

"Regular training at frequent intervals should be there instead of one-time training." {Science teacher School 3, female, age 31}.

"Currently, the training given to our school teachers is not much use unless the training is repeated at frequent intervals." {Science teacher School 2, female, age 40}.

Theme 5: Emerging issues 

*Code 12: Mobile and Social Media Use* 

The issue was highlighted during the visit to urban schools. The teachers informed me that the problem of social media addiction has not risen as such till now. However, given the present conditions, school students can soon get addicted to social media due to more online class scheduling. 

"Since the school is located in a rural area, the students do not possess electronic gadgets, so mobile user behaviour is not rampant. However, with the current situation, where all are requested to use mobile phones extensively, this behaviour can change "{Principal School 2, female, age 50}.

"During the lockdown, the use of mobile phones among children and vulnerable to social media has increased. This issue must be addressed simultaneously as a part of life skills education." {Science teacher School 3, female, age 31}.

## Discussion

Our study findings revealed that participants had less knowledge about the WHO HPS framework and local government policies. The participants perceived health promotion as limited to organizing annual health camps for school children. The Government of India has published operational guidelines for school health programs focusing on health promotion, health screening, immunization, and non-emergency services [8].​ Though they all concurred that some health-related activity was going on in the schools, they were unaware of the guidelines. The Government must ensure the effective dissemination of operational guidelines among all stakeholders for successful school health promotion. 

The issues identified as challenges to the implementation of the HPS framework were inadequate funding for the maintenance of sanitation and first aid box in schools, lack of interactive and repetitive health-related training for teachers, lack of supplementation of micronutrients like calcium and vitamins, inconsistent teachers recruitment policy affecting day-to-day activity, poor coordination with the local community, partial implementation of tobacco-free schools and outdated method of teaching science syllabus. 

During our assessment, the teachers revealed there was no provision for sanitary workers' exclusive fund availability for cleaning the toilets and maintaining cleanliness in the environment. The school receives ₹50,000 as an annual maintenance fund which is grossly inadequate. Teachers feel that a three-time increase in fund allocation is required for school maintenance alone. A similar study in England by Jessiman et al. identified financial constraints, a limited understanding of health constraints, and a narrow focus on academic excellence ​[14]. ​However, the range of issues and priorities vary in both contexts. The health issues in England were predominantly substance misuse and violence, while in Odisha's context, it was a mix of infectious, nutritional, and mental health issues. However, both cases gave financial priority to educational and academic outcome-related issues. 

The teachers expressed more interactive and repetitive health-related training from the Government for health-related issues. Currently, the training is held inconsistently and is a one-off affair. In our assessment, we learnt that the teachers preferred offline training over online and repetition of training. The study was done in Brazil by Gracinio et al., who also reported similar needs from school teachers requiring more guidance from experts for health-related issues [15]. While the teachers in both contexts preferred to have support from professionals for health promotion, the offline support mode over online was reflected in our study, probably due to the flooding of a mixture of verified and unverified information through social media ​[15].

Ideally, all the school teachers, including non-teaching staff, can be trained to provide first aid and selected school students. Our qualitative assessment identified that the first-aid boxes were not adequately maintained and contained expired medicines, and many teachers were unaware of the first-aid box use. 

The participants expressed inconsistent recruitment policies of Governments as another reason for not concentrating on health-related activities and less productivity. Filling vacancies on time will ensure the smooth functioning of the school without any compromises on health. The parliamentary departmental standing committee members have also reflected on this thought ​[16]. This has been a perennial issue throughout the state, and the parliamentary committee emphasized the need for timely utilization of funds for quality improvement and service delivery. 

In our assessment, the teachers commented that the science syllabus in Odia medium is difficult to comprehend as it has more traditional and orthodox words than commonly used colloquial words. This makes it challenging for teachers to teach.

There was broken intersectoral coordination between schools and local bodies. Only a few schools reportedly had a meaningful collaboration with the NGOs. The school headmasters, who had an experience of more than 15 years and worked with at least three different schools, explained that the cooperation from the Local body members depends on his interest, educational status, and familiarity with the public. However, it must be noted that some of the school principals should also take a more proactive role in establishing a meaningful school and community partnership. A similar study in Cambodia by Heesch et al. also revealed the need for more partnerships with the local community [17]. The study points out that collaboration with organizations and individuals beyond local villages is needed to secure funding and technical expertise. The Government of Odisha also introduced the "Mo Schools" initiative to rope in the alumni for voluntary funding, technical expertise, and mentoring of the students. However, village schools do not support such an extensive alumni network apart from the schools in urban areas. ​[17,18]

The local body members and school administration cooperation are vital in ensuring tobacco-free guidelines.​The Government of Odisha also needs to proactively establish cooperation with the Department of Mass Education and the Ministry of Panchayati Raj at the state and district levels [19].

Mobile and social media usage, which has been slowly increasing among students in the last few years, will increase exponentially in the upcoming years. Studies reported an impact on sleep quality, anxiety-related issues, and poor academic achievement for students due to increased mobile usage. Hence, the long-term impact of online classes should be reviewed with mental health concerns ​[20].

The Government of Odisha needs to allocate adequate fund allocation to the school management committee and enable autonomous decision-making powers to cater to the sanitation needs of the schools. Ensuring optimal teacher strength for effective functioning is essential to ensure the welfare of the children. Imparting health-related training to school teaching and non-teaching staff at frequent intervals and enabling inter-sectoral coordination between the school administration and the local body members, health authorities, and NGOs could help overcome the challenges of the implantation issues in health-promoting schools. 

The study is the first of its type done in government schools of Odisha. The qualitative approach provides a platform for policymakers to look into issues specific to the locality by exploring the challenges associated with implementation, as WHO-HPS is a constantly evolving theme.

Limitations

The school selected comprised only government schools, and private schools were not included. Prominent stakeholders of the health-promoting school frameworks, like parents, school students, and Local body leaders, could not be interviewed for the study due to the COVID-19 situation. The psychosocial component of the school is not included in the interview guide. The personal health habits of the children were not elicited in the study due to COVID-19-related restrictions.

## Conclusions

All the participants acknowledged the importance of promoting health in schools. However, their idea of health promotion is mainly restricted to organizing health camps. The participants perceived that the Government of Odisha gives very little importance to health promotion. The respondents did not have much idea about the World Health Organization Health Promoting Schools framework. Health services in the form of Weekly iron-folic acid supplementation, deworming, and immunisation services were present in the schools. However, inadequate fund allocation, less frequent health-related training, sub-optimal teacher strength, and lack of inter-sectoral coordination were reported to be important challenges in implementing health-promoting school frameworks by school administrators and teachers. The Government needs to sensitize all the stakeholders regarding the idea of health-promoting schools based WHO framework.

## Appendices

**Table 3:** Coreq checklist guidelines for qualitative research

No. Item	Guide questions/description	Reported on Page #
Domain 1: Research team and reﬂexivity		
Personal Characteristics		
1. Inter viewer/facilitator	Which author/s conducted the inter view or focus group?	Page 2-3 (First author)
2. Credentials	What were the researcher’s credentials? E.g. PhD, MD	Page 2-3 (MD)
3. Occupation	What was their occupation at the time of the study?	Page 2-3 (Junior Resident)
4. Gender	Was the researcher male or female?	Page 2-3 (Male)
5. Experience and training	What experience or training did the researcher have?	Page 2-3 (Undergone qualitative training)
Relationship with participants		
6. Relationship established	Was a relationship established prior to study commencement?	Page 3 (Established through google meet)
7. Participant knowledge of the interviewer	What did the participants know about the researcher? e.g. personal goals, reasons for doing the research	Page 2-3 (Reasons for doing research)
8. Interviewer characteristics	What characteristics were reported about the inter viewer/facilitator? e.g. Bias, assumptions, reasons and interests in the research topic	Page 2-3 (Intresets in research topic)
Domain 2: study design		
Theoretical framework		
9. Methodological orientation and Theory	What methodological orientation was stated to underpin the study? e.g. grounded theory, discourse analysis, ethnography, phenomenology, content analysis	Page 3 (Grounded theory)
Participant selection		
10. Sampling	How were participants selected? e.g. purposive, convenience, consecutive, snowball	Page 3 (Purposive)
11. Method of approach	How were participants approached? e.g. face-to-face, telephone, mail, email	Page 3 (face-to-face)
12. Sample size	How many participants were in the study?	Page 3 (Nine)
13. Non-participation	How many people refused to participate or dropped out? Reasons?	Page 3 (None)
Setting		
14. Setting of data collection	Where was the data collected? e.g. home, clinic, workplace	Page 3 (Workplace)
15. Presence of non-participants	Was anyone else present besides the participants and researchers?	Page 3 (None)
16. Description of sample	What are the important characteristics of the sample? e.g. demographic data, date	Page 3 (School teachers and principals)
Data collection		
17. Interview guide	Were questions, prompts, and guides provided by the authors? Was it pilot tested?	3 (yes)
18. Repeat interviews	Were repeat interviews carried out? If yes, how many?	No
19. Audio/visual recording	Did the research use audio or visual recording to collect the data?	No
20. Field notes	Were ﬁeld notes made during and/or after the interview or focus group?	Page 3(Yes)
21. Duration	What was the duration of the interviews or focus group?	Page 3 (25 to 40 minutes)
22. Data saturation	Was data saturation discussed?	Page 3 (yes)
23. Transcripts returned	Were transcripts returned to participants for comment and/or correction?	Page 3 (Yes)
Domain 3: analysis and ﬁndings		
Data analysis		
24. Number of data coders	How many data coders coded the data?	Page 3 (Three)
25. Description of the coding tree	Did authors provide a description of the coding tree?	No
26. Derivation of themes	Were themes identiﬁed in advance or derived from the data?	Page 3 (derived)
27. Software	What software, if applicable, was used to manage the data?	Page 3 (Quirkos)
28. Participant checking	Did participants provide feedback on the ﬁndings?	No
Reporting		
29. Quotations presented	Were participant quotations presented to illustrate the themes/ﬁndings? Was each quotation identiﬁed? e.g. participant number	Page 5-7 (Yes)
30. Data and ﬁndings consistent	Was there consistency between the data presented and the ﬁndings?	Page 5-7 (Yes)
31. Clarity of major themes	Were major themes clearly presented in the ﬁndings?	Page 5-7 (yes)
32. Clarity of minor themes	Is there a description of diverse cases or discussion of minor themes?	Page 7 (yes)
